# Magnetic resonance fingerprinting for the whole knee articular cartilage assessment using automated pipeline

**DOI:** 10.1007/s00330-025-11825-5

**Published:** 2025-07-15

**Authors:** Diana Sitarcikova, Veronika Janacova, Malina Gologan, Barbara Hristoska, Martijn A. Cloos, Pavol Szomolanyi, Siegfried Trattnig, Vladimir Juras

**Affiliations:** 1 Department of Biomedical Imaging and Image-Guided Therapy, Medical University Vienna, Vienna, Austria; 2 Donders Centre for Cognitive Neuroimaging, Donders Institute for Brain, Cognition and Behaviour, Radboud University, Nijmegen, The Netherlands; 3 Department of Imaging Methods, Institute of Measurement Science, Slovak Academy of Sciences, Bratislava, Slovakia; 4 CD Laboratory for MR Imaging Biomarkers (BIOMAK), Vienna, Austria; 5 Institute for Clinical Molecular MRI in the Musculoskeletal System, Karl Landsteiner Society, Vienna, Austria

**Keywords:** Magnetic resonance fingerprinting, Cartilage, Relaxometry, T2 mapping, Segmentation

## Abstract

**Objectives:**

To evaluate the feasibility of a prototype MRF sequence in an automated pipeline for T2 extraction of knee cartilage, and to compare it to the same procedure with a conventional T2 mapping sequence.

**Materials and methods:**

Seventeen healthy volunteers and twenty patients with a focal cartilage damage ICRS grade I–III diagnosed via morphological MRI underwent knee MRI examination, including a prototype MRF sequence, a conventional multi-slice multi-echo (MSME) T2 mapping sequence and double-echo steady-state sequence (DESS). Automated cartilage segmentation with a subsequent automated pipeline for T2 extraction from both T2 maps was performed. The methods were compared via correlation analysis. Test–retest analysis was performed on 5 healthy volunteers and evaluated with the intra-class correlation coefficient (ICC). Additionally, the National Institute of Standards and Technology (NIST) phantom was scanned to compare the methods.

**Results:**

On average, the MSME method yielded T2 values 12.4 ms higher than MRF in the phantom and 17.3 ms and 16.3 ms higher in healthy volunteers and patients, respectively. The T2 values correlated very strongly in phantom (*r* = 0.998, *p* < 0.001) and in pooled in vivo analysis (*r* = 0.819, *p* < 0.001 and *r* = 0.865, *p* < 0.001 in volunteer and patient group, respectively), but moderately to strongly in global regions (femoral, patellar and tibial). The reliability of the pipeline was excellent for both methods (ICC from 0.823 to 0.958 for MRF and ICC from 0.863 to 0.932 for MSME).

**Conclusions:**

MRF T2 mapping in the knee cartilage in combination with automatic segmentation is feasible and reliable.

**Key Points:**

***Question*** *Quantitative MRI suffers from long acquisition and post-processing times, reducing its feasibility for routine clinical use in knee articular cartilage applications.*

***Findings*** *T2 mapping via MR fingerprinting in combination with automatic segmentation and T2 extraction procedure in knee articular cartilage performs reliably and time-efficiently.*

***Clinical relevance*** *The proposed combination of methods is suitable for further investigation and characterization of cartilage disorders. The improved time efficiency and high accuracy, together with the elimination of the need for manual input, represent a significant advancement toward clinical translation.*

**Graphical Abstract:**

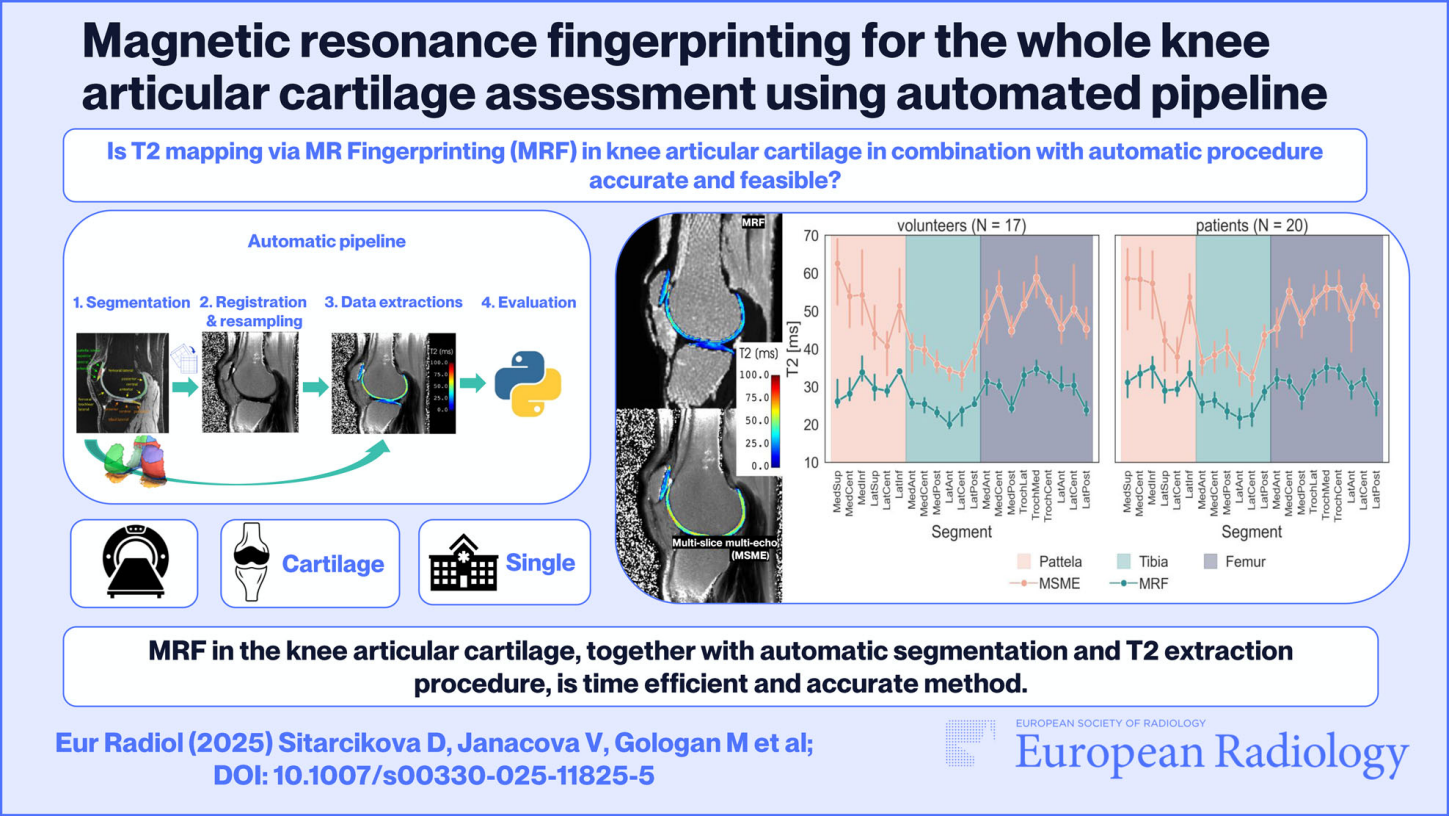

## Introduction

Quantitative MRI is an objective, non-invasive method for the detection and monitoring of pathology, including the knee articular cartilage [1]. In particular, the transverse relaxation time (T2) is a promising MR biomarker, reflecting the water content and organization of the collagen matrix [2], capable of detecting the early stages of osteoarthritis before the onset of morphological changes.

Conventional quantitative MRI techniques are relatively slow and are able to measure only a single parameter at a time. MR fingerprinting (MRF) is a novel state-of-the-art technique that can overcome these limitations [3]. It has been drawing attention due to its speed, accuracy, robustness to artifacts and simultaneous multi-parameter mapping, and was used in a number of clinical applications (e.g., brain [4], abdomen [5], cardiac [6], renal [7] or skeletal muscle [8]). The principle of MRF is to vary the parameters of the acquisition sequence, resulting in unique signal evolution of different tissues. A dictionary with simulated signal evolutions for different tissue and B1 parameters is computed. The acquired signals are then compared to simulated signals, and the best match is assigned to each voxel, which reflects a fingerprint of the tissue properties for the individual patient [3].

ROI definition is the first step in quantitative tissue characterization. Articular knee cartilage is a relatively thin, complex-shaped anatomical structure, which makes its manual delineation tedious. Moreover, manual segmentation is subject to intra- and inter-rater variability, which altogether hinders its clinical translation [9]. Automated procedures are therefore an essential step towards clinical utilization of quantitative MRI. Over the last decades, a number of different automatic or semi-automatic segmentation algorithms of knee articular cartilage have been developed [10]. Fripp et al proposed a model-based segmentation algorithm [11] and reported high accuracy. Model-based segmentation algorithms are popular because of their flexibility, as they can be applied to images with varying resolution, contrast, and signal-to-noise ratio without requiring extensive retraining [9]. This approach is implemented in the MR Chondral Health software.

Therefore, the goal of this study was to evaluate the feasibility of a prototype MRF sequence in an automated pipeline for automatic T2 extraction of the cartilage and compare it to the same procedure with a conventional T2 mapping sequence.

## Materials and methods

This prospective study was approved by the institutional review board (EK1235/2017), and each participant gave written informed consent. The prototype MRF sequence was first evaluated for its accuracy in a phantom measurement. Subsequently, a cohort consisting of healthy volunteers and patients was recruited for knee MRI examination, and the data were analyzed with an automatic post-processing pipeline.

### Study design and study subjects

The MRF sequence was first compared to the conventional MSME sequence in a system phantom developed by the National Institute of Standards and Technology (NIST) [12]. The T2 array of the phantom was used for the study. The array consists of 14 samples with exponentially increasing T2 values in the range of ~5 to ~600 ms.

The study population included a series of consecutively recruited healthy volunteers (scanned between August 2022 and October 2024) with no pain and no history of cartilage damage, and patients (scanned between March 2023 and September 2023) suspected of having focal cartilage damage, ICRS grade I–III diagnosed via morphological MRI.

### MRI examination

All measurements were performed on two 3-T MR scanners using a Tx/Rx 15-channel knee coil (Prisma for patient measurements and PrismaFit for healthy volunteer and phantom measurements, Siemens Healthineers AG) for the knee measurement, and a 20-channel head/neck coil for phantom measurement. The measurement protocol for the study consisted of a prototype MRF sequence with radial readout based on [13], designed to produce proton density, T1, T2, and B1+ relaxation maps. As proposed by Sharafi et al, using a similar MRF sequence, 2 shots were used for the acquisition [14]. A conventional MSME sequence optimized for cartilage T2 mapping evaluation (MapIt, Siemens Healthineers AG) was acquired for comparison. For the knee MRI examinations, double-echo steady-state sequence (DESS) was acquired for automatic cartilage segmentation, and standard, fat-suppressed turbo-spin-echo proton density (FS TSE PD) sequences were acquired for radiological cartilage evaluation. Parameters of the sequences are listed in Table 1. All parametric maps were calculated in-line on the scanner.

**Table 1:** Parameters of the sequences

	MSME	MRF	DESS
Orientation	Sagittal	Sagittal	3D isotropic
TR (ms)	1890	7.5	14.1
TE (ms)	13.8	3.5	5
ETL	8	1	2
FA (°)	90	0–60	25
ST (mm)	4	4	0.6
matrix	384 × 384	192 × 192	256 × 256
FOV	159 * 159	240 * 240	160 * 160
Number of slices	16	16	160
Total time (min:sec)	6:23	4:50	5:58

*TR* repetition time, *TE* echo time, *ETL* echo-train-length, *FA* flip angle, *ST* slice thickness, *FOV* field-of-view, *MSME* multi-slice mutli-echo, *MRF* magnetic resonance fingerprinting, *DESS* double-echo steady-state

The reliability of the MRF sequence was assessed with test–retest measurement, which was performed on a subset of five healthy volunteers during a single session. Between the consecutive measurements, the subjects were taken out of the magnet, got up from the measurement table and were laid down again.

### Image analysis

For the NIST phantom analysis, the slice intersecting through the middle of the T2 array was selected. Circular ROIs were manually drawn inside each sample, and mean T2 values were extracted.

In the knee measurements, model-based automatic segmentation of the cartilage [11] was performed on the DESS images using MR ChondralHealth v3.1 (research package, Siemens Healthineers AG), dividing the cartilage into 21 segments (6 patellar, 6 tibial and 9 femoral). All segmentations were reviewed by a research fellow with 2 years of experience in cartilage imaging (D.S.).

**Fig. 1:**
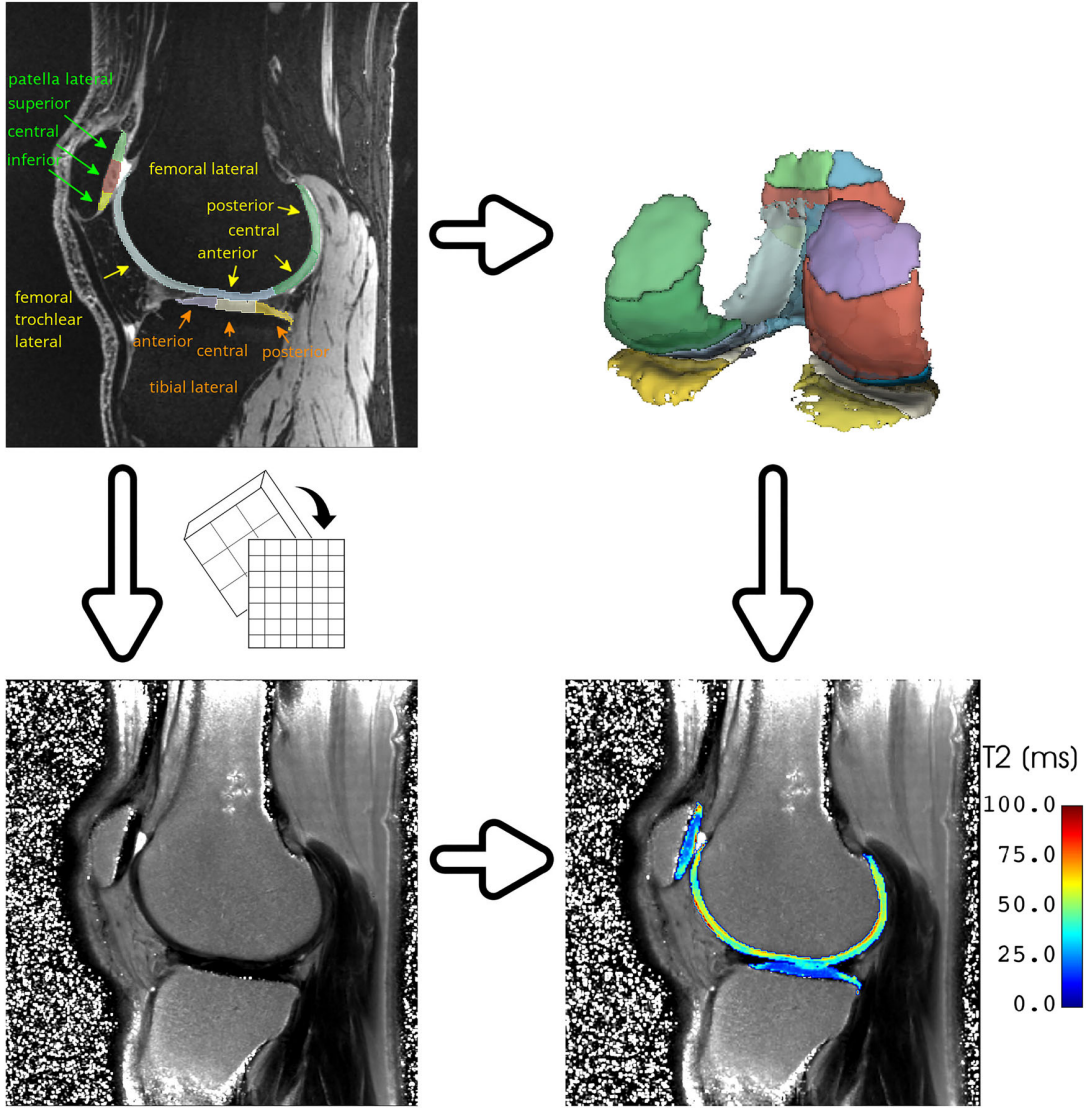
Steps of the automated pipeline. (Top row) DESS images were used for automatic segmentation of the cartilage in the MR ChondralHealth software. Subsequently, DESS images, segmentation and MSME and MRF T2 maps were loaded to 3D Slicer. Both T2 maps were registered and resampled to DESS images, and segmentation was overlaid on the resulting images. Extreme values were masked out, and mean T2 values for each segment were extracted for further analysis

Subsequently, the DESS images, MSME and MRF T2 maps and segmentation were post-processed with an automated pipeline in the 3D Slicer image computing platform v5.1 [15]. The pipeline first loaded the images and segmentation. Then, both relaxation maps were registered and resampled to DESS images and segmentation dimensions. This was accomplished with the General registration (BRAINS) module. DESS was selected as fixed volume, T2 map as moving volume and rigid transformation type, with 20% of voxel samples used for registration. Segmentation was overlaid on each registered and resampled T2 map, and prior to mean T2 value extraction of each segment, values below 5 ms and above 150 ms were masked out. Those extreme values were presumed to belong to synovial fluid, bone, chemical shift artifact, or poor fit. The results of this automatic process were visually inspected, and in case of imprecise alignment of segmentation and cartilage, the segmentation was manually translated in the sagittal plane in resampled volumes by 1–2 pixels. Steps of the automated pipeline are depicted in Fig. 1.

**Fig. 2:**
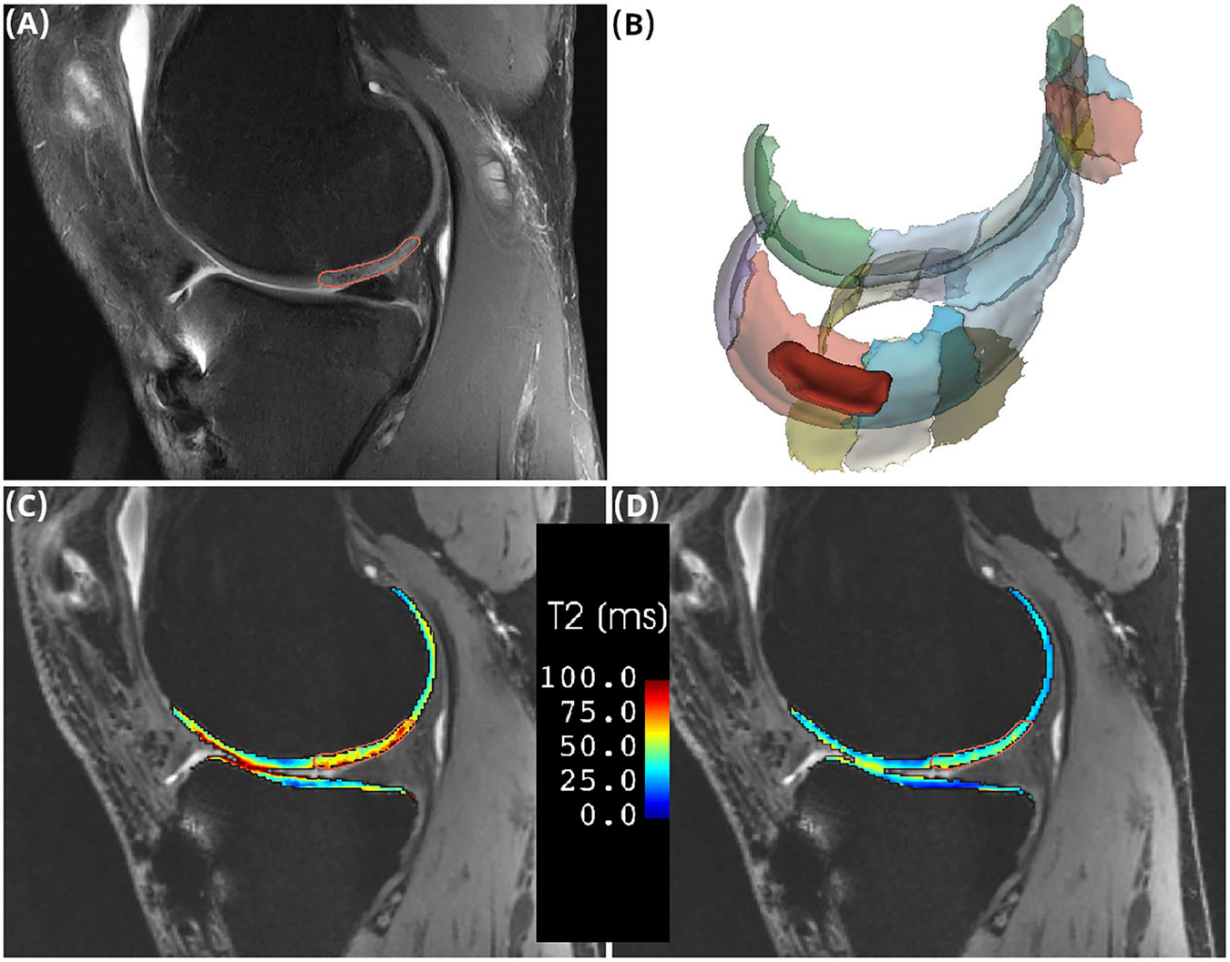
FS TSE PD image showing a cartilage lesion identified by an experienced MSK radiologist (**A**), guiding lesion segmentation within the automatic cartilage segmentation mask (**B**). Corresponding slices of MSME (**C**) and MRF (**D**) cartilage T2 maps with the lesion

FS TSE PD images were reviewed by an experienced MSK radiologist (S.T. > 30 years of experience) to identify the lesions. These lesions were then segmented within the boundaries of the automatic segmentations, and mean T2 relaxation times were extracted from the resampled, registered, and masked T2 maps (Fig. 2).

### Statistical analysis

All statistical computations were performed in Python (version 3.7.0). Data were presented as counts for categorical variables, mean ± standard deviation (SD) for continuous variables if data were normally distributed, and median (1st quantile, 3rd quantile) if data were not normally distributed. The Shapiro–Wilk test was used to assess data normality.

Pearson's correlation coefficient was calculated to assess the strength of the linear relationship between the methods. Bland–Altman analysis was performed to assess bias between the two methods. A paired *t*-test was used to test the statistical significance of the difference between the two methods if differences were distributed normally; otherwise, the Wilcoxon signed-rank test was used. Test–retest measurement was analyzed with two-way mixed effects, single-measurement, consistency intra-class correlation coefficient ICC (3,1).

## Results

### Phantom measurements

**Fig. 3:**
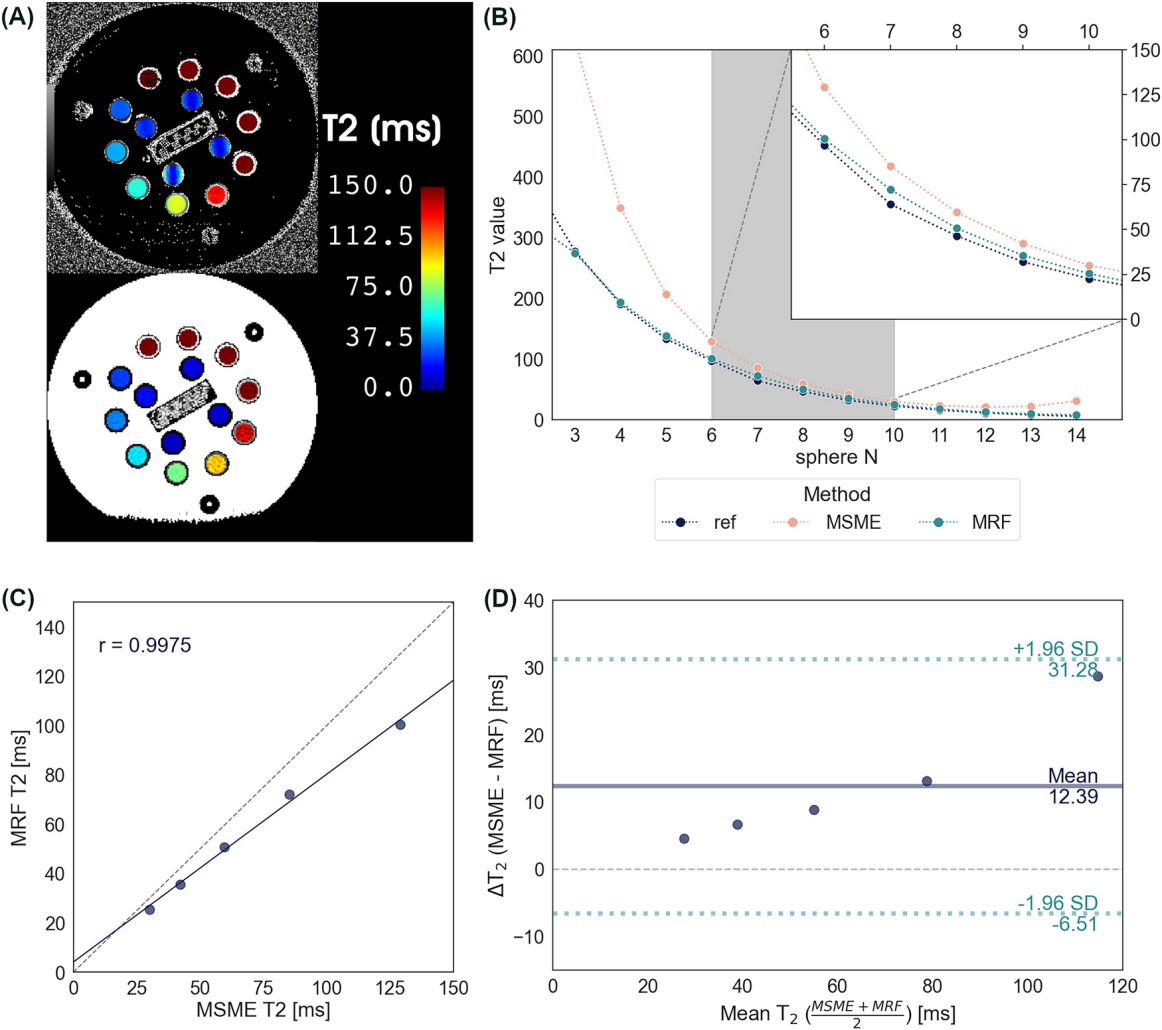
**A** Representative MSME (top) and MRF (bottom) T2 maps. **B** Mean MSME, MRF, and reference T2 values measured in individual samples of the NIST phantom. Gray area marks the analyzed range of the NIST phantom, which corresponds to cartilage T2 values. **C** Correlation and (**D**) Bland–Altman plot between MSME and MRF T2 values from samples No. 6 to 10

Representative MSME and MRF T2 maps are depicted in Fig. 3A, and corresponding mean T2 values of each sphere are shown in Fig. 3B, together with the reference T2 values given by the manufacturer. Since the MRF T2 map is capped at ~330 ms, spheres No. 1 and 2 are not shown.

Only spheres No. 6 to 10, which fall into the expected range of T2 values in the healthy and damaged cartilage, were further included in the analysis. MRF and MSME T2 values demonstrated a strong linear relationship (*r* = 0.998, *p* < 0.001, Fig. 3C), but there was a mean bias of 12.4 ms between MSME and MRF in the analyzed range, and the bias was linearly increasing with increasing T2 value (Fig. 3D).

### In vivo knee measurements

All recruited subjects provided analyzable data and were included in the final analysis. There were 17 volunteers (8 male, 9 female, with a mean age of 33.0 years) and 20 patients (15 male, 5 female, with a mean age of 35.23 years) scanned. Two patients underwent examination on both knees; therefore, 22 patient knees were scanned in total. After revision by the experienced radiologist, 20 of patients’ knees had at least one focal cartilage damage, with a total of 35 cartilage lesions identified (of which 17 were femoral, 4 trochlear and 14 patellar).

The automatic segmentation was successful in all subjects, but there was manual adjustment necessary in five cases. After the automatic post-processing pipeline, the segmentation was slightly misaligned with cartilage in 4 MSME resampled T2 maps and in 4 MRF resampled T2 maps. The proportion of presumed aberrant T2 values (i.e., those falling outside the predefined range of 5–150 ms) was 2.6 ± 4.3% for MRF and 2.3 ± 4.0% for MSME. There was no statistically significant difference between the two methods (*p* = 0.094).

**Fig. 4:**
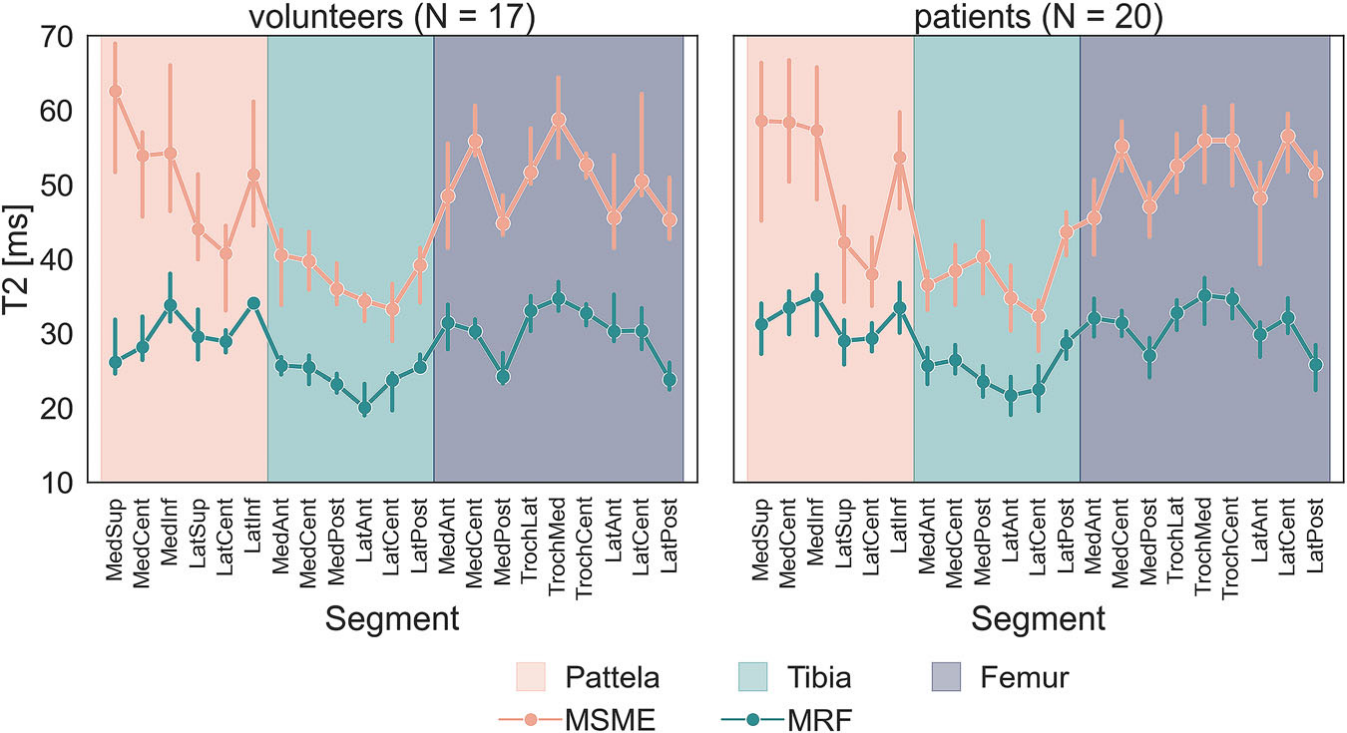
Mean T2 values with inter-quartile range of all segments in the volunteer and patient groups measured by the MSME and MRF methods

The median T2 value from all regions was 49.9 and 33.0 ms for MSME and MRF methods, respectively, in the volunteer group, and 51.0 and 34.3 ms for MSME and MRF methods, respectively, in the patient group. Table 2 and Fig. 4 summarize mean or median values for each segment in each group. The mean differences between methods are also listed in the table, and the difference between methods was statistically significant in all segments (*p* < 0.001). The mean T2 value of all 35 lesion regions was 56.7 ± 10.9 and 38.6 ± 7.7 ms for MSME and MRF methods, respectively. The mean lesion volume was 0.77 ± 0.46 mL, which was obtained concurrently with the T2 extraction. As expected from phantom measurements, the MSME method overestimated T2 values, and the overestimation was higher in segments with higher T2 values.

**Table 2:** Mean or median MSME and MRF T2 values in volunteer and patient groups

	Group	Volunteer			Patient		
	method	MSME	MRF		MSME	MRF	
Global region	Sub-region	Mean/median ± sd/quartiles	Mean/median ± sd/quartiles	$\bar{\Delta }$ T2 (MSME-MRF)	Mean/median ± sd/quartiles	Mean/median ± sd/quartiles	$\bar{\Delta }$ T2 (MSME-MRF)
Patellar sub-divided cartilage	MedSup	60.7 ± 10.5	32.3 ± 6.1	28.4	60.3 ± 11.7	38.6 ± 8.9	21.7
	MedCent	55.2 ± 9.7	33.3 ± 4.9	21.9	59.9 ± 10.8	40.2 ± 7.6	19.7
	MedInf	60.9 ± 12.3	42.5 ± 8.0	18.4	60.1 ± 14.0	39.1 (35.2, 46.5)	18.3
	LatSup	50.2 ± 9.5	32.7 ± 4.7	17.5	41.9 (37.7, 53.1)	32.6 ± 7.2	13.7
	LatCent	43.0 ± 8.1	31.1 ± 4.0	11.8^a^	42.6 ± 7.9	32.5 ± 5.3	10.1
	LatInf	55.7 ± 10.4	36.4 (34.3, 40.3)	16.6	55.8 ± 12.1	39.0 ± 7.1	16.7
	Whole patella	54.3 ± 11.7	34.4 (30.2, 38.4)	19.1	53.1 (43.0, 62.6)	36.3 (31.0, 42.9)	16.7^a^
Tibial sub-divided cartilage	MedAnt	45.6 ± 5.8	30.5 ± 3.6	15.2	38.1 (37.0, 42.0)	27.8 (25.4, 31.8)	12.3
	MedCent	44.1 ± 6.9	28.2 (27.0, 30.4)	14.5	42.9 ± 6.5	30.5 ± 5.2	12.4
	MedPost	41.9 ± 6.4	26.7 (26.4, 28.9)	14.0	45.4 ± 6.6	28.9 ± 4.9	16.5
	LatAnt	41.9 ± 3.7	25.7 ± 3.7	16.2	40.2 ± 7.2	25.7 ± 4.1	14.5
	LatCent	35.2 ± 4.9	25.1 ± 3.2	10.1	35.6 ± 5.9	24.9 ± 4.4	10.6
	LatPost	41.9 ± 5.1	29.6 ± 2.6	12.3^a^	46.9 ± 5.0	33.5 ± 6.9	13.5
	Whole tibia	41.8 ± 6.3	27.7 (25.7, 30.1)	13.7	40.6 (36.7, 48.1)	28.2 (25.0, 31.5)	13.3
Femoral sub-divided cartilage	MedAnt	52.8 ± 7.6	37.4 ± 4.2	15.4	49.5 ± 7.4	37.8 ± 5.2	11.6
	MedCent	60.8 ± 5.3	36.5 ± 4.1	24.3	58.2 ± 5.4	36.2 ± 4.4	22.0
	MedPost	48.3 ± 5.5	28.9 ± 3.1	19.5	48.3 (44.0, 51.2)	28.8 (26.5, 35.9)	18.4
	TrochLat	55.4 ± 6.0	40.1 ± 4.5	15.2	55.0 ± 5.8	39.8 (37.2, 42.2)	15.5
	TrochMed	60.9 ± 6.4	41.9 (40.8, 42.8)	18.5	57.6 ± 7.5	42.3 (38.2, 44.1)	15.4
	TrochCent	54.3 ± 3.7	37.7 ± 2.8	16.6	57.9 ± 8.3	39.9 ± 5.8	18.0
	LatAnt	53.8 ± 9.8	39.8 ± 6.7	13.9	50.8 ± 10.2	35.3 ± 6.6	15.5
	LatCent	56.4 ± 8.0	33.7 (32.5, 42.4)	20.1	59.6 ± 7.1	37.4 ± 5.4	22.2
	LatPost	51.0 ± 5.8	29.3 ± 3.7	21.7	55.3 ± 5.8	32.0 ± 4.4	23.3
	Whole femur	54.9 ± 7.6	36.4 (32.0, 41.3)	18.4	54.7 ± 8.2	36.8 (32.8, 40.8)	18.0
	Whole cartilage	49.9 (43.7, 57.0)	33.0 (28.4, 38.2)	17.3^a^	51.0 (42.2, 57.9)	34.3 (28.9, 39.6)	16.3^a^

T2 values were calculated for each of the 21 segments, as well as the global regions and whole cartilage. Mean difference and statistical significance of the differences are also listed

*Med* medial, *Lat* lateral, *Sup* superior, *Cent* central, *Inf* inferior, *Ant* anterior, *Post* posterior, *MSME* multi-slice multi-echo, *MRF* magnetic resonance fingerprinting, *DESS* double-echo steady-state

^a^ Wilcoxon signed-rank test was used to test differences

**Fig. 5:**
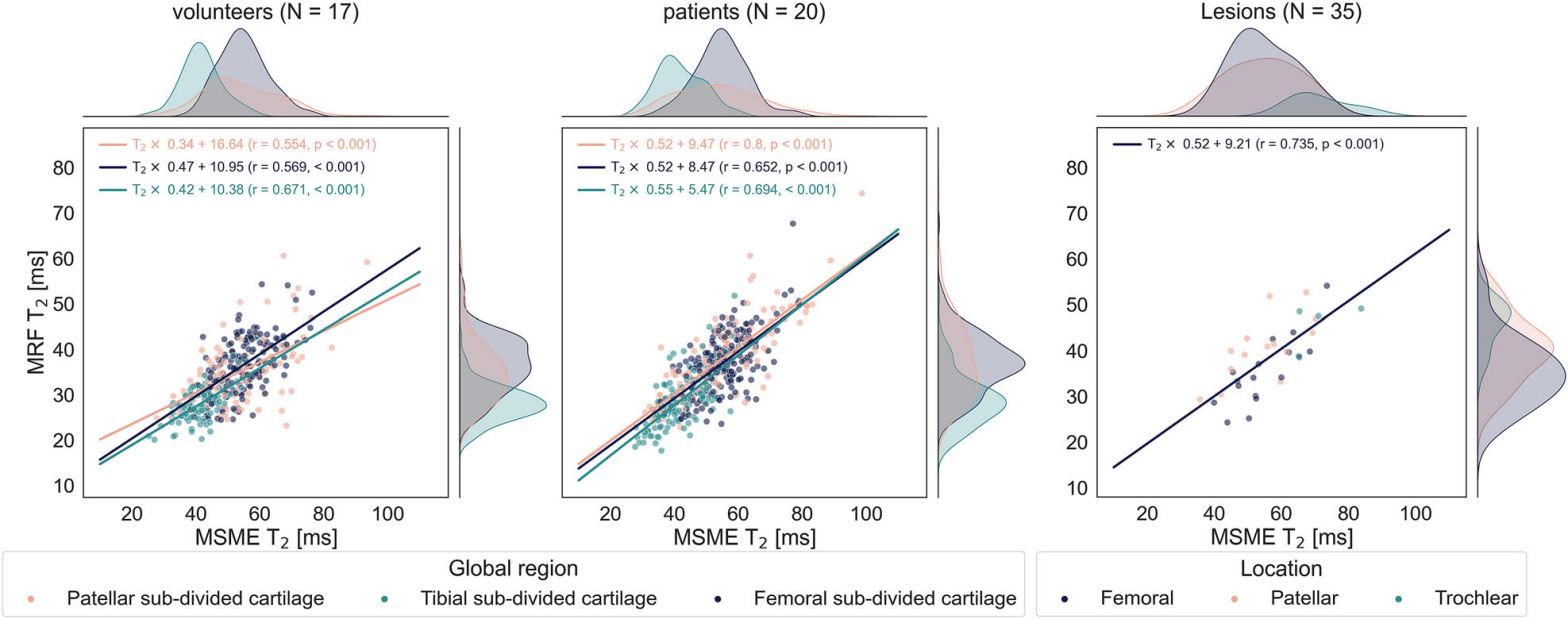
Scatterplots depicting linear correlation between T2 values measured by MSME and MRF for the volunteer and patient groups

Pooled correlation between MRF and MSME from all segments was *r* = 0.819 (*p* < 0.001) and *r* = 0.865 (*p* < 0.001) in the volunteer and patient group, respectively. MRF and MSME T2 values exhibited a moderate to strong linear relationship in global sub-regions of the cartilage. The correlation between T2 values from lesion segments was *r* = 0.735 (*p* < 0.001) (Fig. 5).

**Fig. 6:**
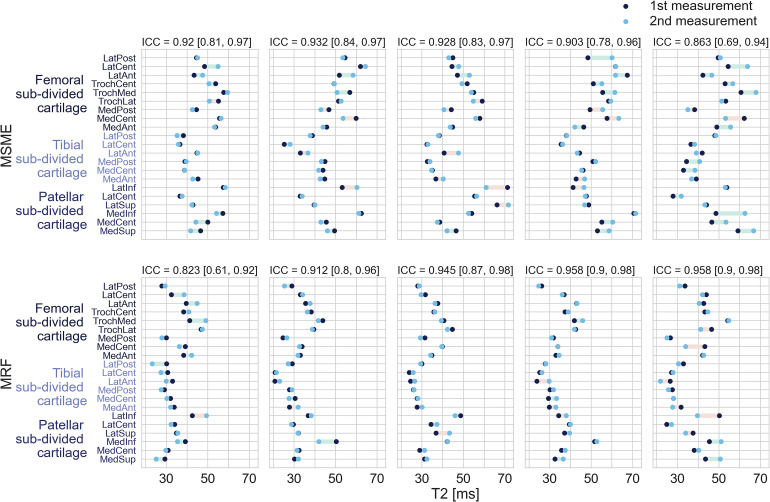
Lollipop plot depicting the results of the first and second measurements in each subject

The results of the test–retest measurements in five volunteers are depicted on Fig. 6. High degree of reliability was found between the two measurements in all subjects in both methods.

## Discussion

In this paper, an automated assessment of whole cartilage T2 relaxation times from the MRF sequence was performed and compared to an automated assessment of whole cartilage T2 values from the conventional MSME T2 mapping sequence. A bias between the two methods was observed in phantom and in vivo measurements, but a moderate to very strong correlation between the methods was revealed.

Our results in phantom showed a systematic bias between MRF and MSME T2 mapping, which was confirmed in in vivo measurements. One of the reasons was probably the limitation of the MSME sequence and its formation of stimulated echoes, which were shown to overestimate the measured T2 values [16]. Nevertheless, the MSME sequence used in our study was optimized for cartilage T2 mapping. The choice of echo times in MSME was set to accurately estimate T2 in the cartilage. Therefore, only spheres with T2 values that are expected in the knee cartilage were selected for analysis in the NIST phantom. On the contrary, the measurement in the phantom indicates that MRF estimates T2 accurately across a much broader range of T2 values, as is evident in Fig. 3B.

The MRF sequence adapted in our study was originally developed for the T1 and T2 mapping of the hip articular cartilage [13]. Sharafi et al extended the sequence by adding a T1rho preparation module to encode T1rho mapping and evaluated the proposed sequence in knee cartilage and phantoms [17]. Recently, they implemented a 3D stack-of-stars MRF sequence with 18 slices for whole cartilage coverage T1, T2, and T1rho mapping with manual segmentation of the cartilage [14]. Kijowski et al documented age-related differences in T2 and T1rho via MRF sequence [18], underlining the sensitivity of MRF.

One of the main challenges of our study was the differently resolved images and segmentation in combination with the complex anatomy of the cartilage. Co-registering and resampling the T2 maps to DESS images, and therefore the segmentation, was shown to have high repeatability when using a 3D triple-echo steady-state sequence for T2 mapping [9]. In our study, the same approach was used; however, the co-registration and resampling were performed with the open-source 3D Slicer’s built-in module for registration, further increasing the reproducibility of our study. We also demonstrated high repeatability with this automated procedure for both MSME and MRF methods.

The T2 relaxation time distribution is not homogeneous throughout the cartilage [19] and depends on the organization of collagen fibrils, which are organized in a zonal manner in the bone-to-surface direction [20]. Moreover, this zonal orientation is different in weight-bearing and non-weight-bearing regions of the cartilage [2]. On top of that, factors like magic angle effects [21], exercise [22, 23] or sex and gender [24] might further influence the T2 values.

Even though the zonal T2 pattern across cartilage thickness cannot be captured with the current resolution of the MRF sequence, the bulk T2 value of individual segments can be compared. Direct comparison of absolute T2 values with the literature is challenging, since the observed T2 value depends on the acquisition method, and moreover, the compartmentalization of the cartilage segmentation is not uniform across studies. However, the relative values of individual regions can be aligned with the literature. Generally, the lowest T2 values were found in tibia regions, being mainly weight-bearing cartilage, which is in line with the reported cartilage T2 values in a number of studies [18, 19, 24, 25]. Patellar cartilage exhibited the greatest spread of T2 values, as the mechanical stress and motion patterns are much broader than in other cartilage compartments, which is also evident in published literature. Surowiec et al performed a T2 extraction from the same 21 segments from an MSME T2 map using manual segmentation, and they reported significant differences in T2 values between some of the segments [19]. Even though we did not perform statistical tests between T2 values from different segments, the MSME T2 values found in our study strongly correlate to the results of Surowiec at al. These findings further validate the accuracy of the automatic pipeline for T2 extraction and its ability to capture the regional variation of T2 in the cartilage, in spite of different sequence parameters and a significantly lower number of acquired slices in our study (16 vs 25). Further consensus can be found when comparing our MSME results to the study by Kaneko et al, who studied the variation of T2 of femoral cartilage with regard to angle to B0 [26]. Femoral cartilage has weight-bearing and non-weight-bearing regions, with differently oriented collagen fibers, and magic angle regions with artificially increased T2 values, which were well illustrated by Kaneko et al [26]. In our study, medial and lateral central regions of the femoral cartilage are the regions mostly affected by the magic angle effect with increased T2 values, and medial and lateral anterior segments of the femoral cartilage are the weight-bearing regions with decreased T2 values. The posterior regions of medial and lateral femoral cartilage were also reported with decreased T2 values within the femoral cartilage [19, 26]. It is worth mentioning that the T2 values found in our study were consistently higher than cartilage T2 values reported in literature [18, 19, 24–26]. This can be attributed to the interpolation step of the post-processing pipeline, where new data points were computed. Even though outlier values were masked out to exclude the influence of other tissues, some residual influence was inevitable.

The MRF T2 values correlated very strongly with MSME values in the overall pooled analysis. The correlation was only moderate to strong between values of individual segments in the global regions. This is expected, since a narrower range of T2 values is analyzed for each global region, containing a much higher number of data points. Moreover, patient data exhibited a stronger correlation compared to volunteer data, because the range of T2 values of patients was broader due to pathologies in the cartilage, altering the T2 values. Some bias was presumably introduced in the co-registration process, since new values were computed in the interpolation step. It remains uncertain whether the correlation coefficient accurately reflected the agreement between methods or was predominantly influenced by the precision of the co-registration process.

From visual inspection of Fig. 4, it can be noticed that MRF reflected regional variations in T2 values, but the medial and lateral anterior regions of femoral and medial superior patellar cartilage exhibited deviations from the expected regional T2 variation. This could be due to the frequent presence of fluid between femoral and tibial cartilage and its partial volume effect in the relatively small resolution of MRF, or this may be attributable to a systematic bias inherent in the processing pipeline.

Our study had some limitations. First, the temperature of the NIST phantom was not directly recorded during the acquisitions. While all measurements were acquired in a single scan session, minimizing temperature variation, this lack of temperature control introduces some uncertainty when comparing our results to the reference T2 values. Second, the relatively low spatial resolution of MRF (6.25 μL voxel size) might reduce sensitivity to very small, focal lesions seen in early osteoarthritis. However, in this study, we have indeed shown the feasibility of detecting elevated T2 in OA cartilage lesions with this resolution using MRF. Third, the automatic segmentation algorithm was not perfect. Even though all segmentations were reviewed, only significant imperfections were addressed through manual correction. Besides that, masking of outliers was also performed to exclude the influence of surrounding tissue and artifacts. Fourth, the effect of partial volume in 2D acquisition was unavoidable, mainly in the trochlear regions of the cartilage. Fifth, no comparison of the volunteer and patient groups was performed. Nevertheless, given the relatively small size of lesions compared to whole cartilage, no significant difference in T2 values would be expected. However, this will be subject to the next step of our study, with targeted region of lesions identification. Sixth, the co-registration and resampling pipeline is subject to imperfections, which could influence the outcomes of our analysis.

In conclusion, MRF T2 mapping in the knee cartilage in combination with automatic segmentation is feasible in healthy volunteers and patients and correlates with the conventional MSME method. Our pipeline’s results are in line with published literature, supporting its validity and applicability.

## Abbreviations

DESS: Double-echo steady-state sequence
ICC: Intra-class correlation coefficient
MRF: MR fingerprinting
MSME: Multi-slice multi-echo
NIST: National Institute of Standards and Technology
